# Probabilistic Error-Corrected Controlled Dense Coding Under Bit-Flip Channels via Auxiliary Particles and Partially Entangled States

**DOI:** 10.3390/e28070778

**Published:** 2026-07-08

**Authors:** Zitong Diao, Jie Tang, Zhaoqi Lei, Huicun Yu, Jiahao Li, Lei Shi, Jiahua Wei

**Affiliations:** Information and Navigation College, Air Force Engineering University, Xi’an 710077, China; diaozitong1176@163.com (Z.D.); 18014102582@163.com (J.T.); leizhaoqi0505@163.com (Z.L.); yhc9426@163.com (H.Y.); quantum_ljh@163.com (J.L.); slfly2012@163.com (L.S.)

**Keywords:** probabilistic dense coding, controlled dense coding, partially entangled channel

## Abstract

Quantum dense coding could be used to transmit two classical bits with one qubit when a maximally entangled state is shared. In realistic channels, entanglement degradation reduces the channel capacity, while bit-flip noise increases decoding errors. To address these issues, we propose a novel probabilistic controlled dense coding protocol that employs the three-qubit repetition code for error correction and an auxiliary qubit for probabilistic decoding. Moreover, this proposed scheme includes a third party to supervise the communication based on a three-qubit entanglement state. The implementation steps of our protocol are presented in detail, and numerical simulations show that it achieves higher average information than dense coding without error correction. The scheme provides a robust solution for quantum communication under noisy conditions and non-maximally entangled state.

## 1. Introduction

Quantum dense coding (QDC) was first proposed by Bennett in 1992 [1] and experimentally realized by Mattle [2]. In the typical dense coding protocol, the sender can transmit two bits of classical information by sending a single qubit to the receiver after sharing a maximally entangled state [3,4]. This protocol surpasses the Holevo limit, achieving a capacity of two classical bits per transmitted qubit [5]. However, the ideal performance of the dense coding scheme relies on maximally entangled states [6], which are vulnerable to decoherence and degrade into partially entangled states over time or distance [7,8]. This degradation reduces the channel capacity, posing a fundamental limitation on practical quantum dense coding [9,10]. Moreover, during the transmission of encoded qubits, channel noise such as the bit-flip errors further disrupts the encoded state [11]. Such noise leads to higher error rates and reduced channel capacity, even with optimal decoding [12,13]. These unavoidable issues in practical quantum communication has motivated the development of mitigation strategies, including the use of classical error-correcting codes to protect quantum states [14,15,16,17,18].

Probabilistic dense coding using Positive Operator-Valued Measure (POVM), first investigated by Hao et al. [19] and Pati [20], enables unambiguous decoding when successful, while deterministic dense coding has also been explored [21]. However, its success probability is fundamentally limited by the degree of initial entanglement [22]. This limitation arises from the non-orthogonality of encoded states when the shared channel is only partially entangled [7,8]. A parallel line of research introduces supervisory control into dense coding. For instance, controlled dense coding using the Greenberger–Horne–Zeilinger (GHZ) state has been studied [23]. Similar control ideas also appear in probabilistic teleportation schemes [24,25,26]. In distributed scenarios involving multiple senders and receivers under non-Markovian noise, Muhuri et al. [27] analyzed the dense coding capacity. At the hardware level, Livingston et al. [28] experimentally demonstrated continuous bit-flip correction using a three-qubit repetition code and stabilizer-based methods, showing a 2.7-fold increase in relaxation time. The three-qubit repetition code has been successfully applied in long-distance quantum communication, including repeater-assisted quantum key distribution [29], encoded repeater chains [30], and scalable quantum network nodes based on nitrogen-vacancy centers [31]. These studies have demonstrated the practical relevance of the code for combating bit-flip errors in realistic long-distance settings. Meanwhile, some pioneering studies in long-distance quantum communication are presented including the preparation of high-dimensional frequency-orbital angular momentum entangled states [32], entanglement scaling in topological phases [33], hierarchical remote state preparation [34], and deterministic remote implementation of quantum operations [35]. These developments further enrich the background of error-corrected quantum communication.

Despite these advances, existing schemes address only isolated aspects. Probabilistic dense coding schemes do not incorporate error correction. Controlled dense coding protocols typically assume ideal channels without noise. Hardware level error correction has not been integrated with probabilistic decoding or control mechanisms. Consequently, a unified framework that combines quantum error correction, supervisory control, and probabilistic decoding to simultaneously address entanglement degradation and bit-flip noise has not yet been proposed.

In this work, we propose a robust protocol that integrates the three-qubit repetition code with probabilistic controlled dense coding. Section 2 briefly reviews the three-qubit repetition code. We establish a typical error-corrected scheme to combat channel noise in Section 3. Building on this foundation, we introduce a controller to enable supervised communication in Section 4. Subsequently, we generalize the protocol to partially entangled states and employ probabilistic decoding to handle the resulting non-orthogonality in Section 5. The performance of this scheme is analyzed in Section 6. Additionally, Section 7 discusses the practical applications and experimental challenges. Section 8 concludes the paper.

## 2. Brief Overview of the Three-Qubit Repetition Code

**Figure 1:**
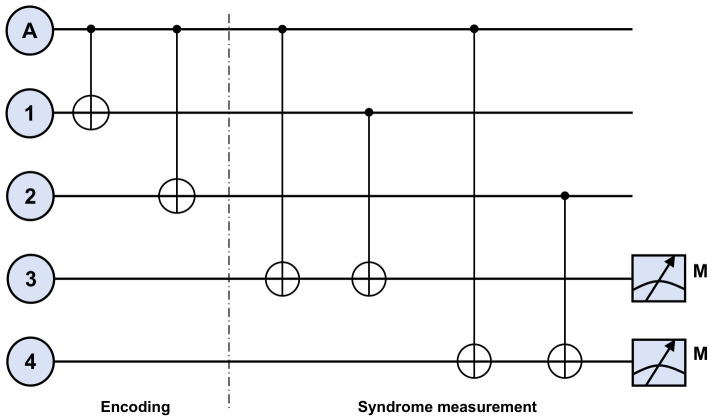
Quantum circuit of the three-qubit repetition code, consisting of an encoding stage and a syndrome measurement stage. The encoding uses two $C_{NOT}$ gates to map a logical qubit to three physical qubits. The syndrome measurement uses two ancillary qubits to measure the stabilizers $Z_{A}Z_{1}$ and $Z_{A}Z_{2}$.

Quantum error correction works by encoding information into a larger Hilbert space so that different errors map the state into mutually orthogonal subspaces, allowing detection without revealing the encoded information [14]. The three-qubit repetition code is a typical example of such a code. It is designed to correct a single-qubit error. In this code, a logical qubit is encoded into three physical qubits as

(1) $$|0\rangle \to |0_{L}\rangle \equiv |000\rangle ,\;|1\rangle \to |1_{L}\rangle \equiv |111\rangle$$

where the three qubits are denoted as A,1,2. The encoding circuit is shown in the Encoding part of Figure 1, where two Controlled-NOT ($C_{NOT}$) gates with the original qubit *A* as the control prepare the redundant state $|000\rangle$ or $|111\rangle$. After transmission through a noisy channel, if at most one error occurs, the receiver can detect it by measuring the stabilizers $Z_{A}Z_{1}$ and $Z_{A}Z_{2}$ [36]. The syndrome measurement circuit is shown in the Syndrome measurement part of Figure 1, using two ancillary qubits 3 and 4 initialized in $|0\rangle$. The two-bit syndrome uniquely identifies the location of the error, and applying an appropriate Pauli gate to the faulty qubit restores the original state.

It can be used in quantum communication scenarios where one type of error dominates [28,29,30,31]. However, it has fundamental limitations. It can correct only one error per encoded block and fails when two or more errors occur. Moreover, it cannot correct both a bit-flip and a phase-flip error at the same time. Despite these limitations, it serves as a useful building block for understanding more powerful quantum error correction codes [17]. 

## 3. The Typical Scheme for Error-Corrected Dense Coding

We begin with the typical dense coding protocol. The sender Alice and the receiver Bob initially share the maximally entangled Bell state

(2) $${|\Psi^{+}\rangle }_{AB}=\frac{1}{\sqrt{2}}{\left(|00\rangle +|11\rangle \right)}_{AB}$$

To send one of the four two-bit messages $\left\{00,01,10,11\right\}$, Alice applies one of the four Pauli operations $\left\{I,\sigma_{x},\sigma_{y},\sigma_{z}\right\}$ to her qubit *A* and sends it to Bob. Bob then performs a Bell-state measurement on the two-qubit system, which allows him to perfectly distinguish the state and decode the two bits of classical information. However, this ideal protocol assumes a noiseless channel. To address bit-flip errors during transmission, Luo et al. proposed a two-party error-correction scheme that uses the three-qubit repetition code and auxiliary particles for error diagnosis under maximally entangled Bell states [37]. The modified procedure is as follows:

**Step 1:** After applying the chosen Pauli operation, Alice encodes her logical qubit into three physical qubits by applying two $C_{NOT}$ gates, to implement the three-qubit repetition code as described in Equation (1).

**Step 2:** Alice transmits the three encoded qubits (A,1,2) through a noisy quantum channel, modeled here as the standard bit-flip channel. Each particle independently traverses a bit-flip channel, where it remains unchanged with probability $p(\frac{1}{2}\leq p\leq 1)$ or undergoes a bit-flip with probability 1−p.

**Step 3:** Upon receiving the physical qubits, Bob performs nondestructive error diagnosis using two auxiliary qubits (3,4) initialized in the state ${|0\rangle }_{3}{|0\rangle }_{4}$. He applies the $C_{NOT}$ gates $C_{NOT}\left(A,3\right),C_{NOT}\left(1,3\right),C_{NOT}\left(A,4\right),C_{NOT}\left(2,4\right)$, where the first index denotes the control qubit and the second the target. The role of these gates is to encode the parity information between qubit pairs into the auxiliary qubits. The gates $C_{NOT}\left(A,3\right)$ and $C_{NOT}\left(1,3\right)$ encode the parity of qubits *A* and 1 into auxiliary qubit 3, flipping it to $|1\rangle$ only when the two qubits differ. This operation is equivalent to measuring the stabilizer $Z_{A}Z_{1}$. Similarly, $C_{NOT}\left(A,4\right)$ and $C_{NOT}\left(2,4\right)$ encode the parity of qubits *A* and 2 into auxiliary qubit 4, thereby measuring $Z_{A}Z_{2}$. The combined state evolves to

(3) $$C_{NOT}\left(A,4\right)C_{NOT}\left(2,4\right)C_{NOT}\left(A,3\right)C_{NOT}\left(1,3\right)\left[{|\psi \rangle }_{A}{|\psi \rangle }_{1}{|\psi \rangle }_{2}{|0\rangle }_{3}{|0\rangle }_{4}\right]$$

The process is equivalent to measuring the stabilizers $Z_{A}Z_{1}$ and $Z_{A}Z_{2}$ without collapse. Bob measures auxiliary qubits (3,4), obtaining a two-bit syndrome $\left(s_{1},s_{2}\right)$ that locates the error [36,38]. If a single bit-flip error is identified, Bob applies the $\sigma_{x}$ operation to the faulty qubit. Provided that at most one error has occurred, this operation deterministically recovers the original encoded state. After error correction, Bob reverses the encoding to obtain the logical qubit. Since qubits 1 and 2 are the same logical state as *A* after encoding, the measurement on the four qubits (A,1,2,B) reduces to a Bell-state measurement on (A,B), allowing Bob to decode the two classical bits.

This protocol protects against any single bit-flip error among the three transmitted qubits, preserving the ideal dense coding capacity of two classical bits per logical qubit. Its success probability is

(4) $$P_{c}=p^{3}+3\left(1-p\right)p^{2},\left(\frac{1}{2}\leq p\leq 1\right)$$

which is greater than or equal to the success probability *p* of the typical protocol without error correction. This establishes a foundational error-corrected dense coding scheme that directly addresses channel noise.

## 4. Controlled Dense Coding with Error Correction

**Figure 2:**
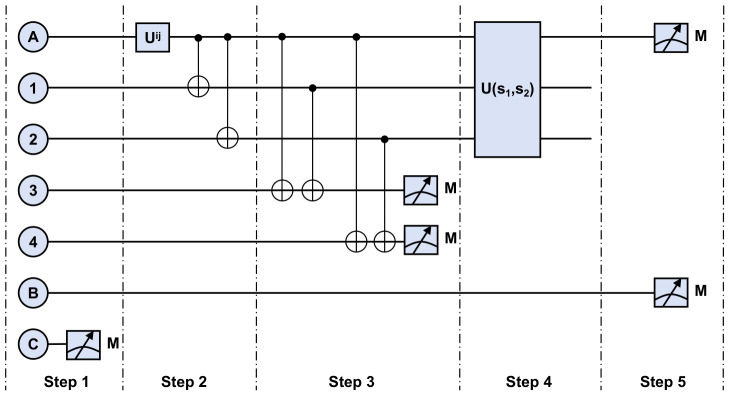
Quantum circuit for our error-corrected controlled dense coding protocol using a maximally entangled GHZ state. The unitary operations $U^{ij}$ (i,j=0,1) applied by Alice correspond to the four Pauli operators: $U^{00}=I$, $U^{01}=\sigma_{z}$, $U^{10}=\sigma_{x}$, $U^{11}=i\sigma_{y}$. Bob’s correction operator $U(s_{1},s_{2})$ is applied according to the error syndrome $\left(s_{1},s_{2}\right)$ obtained from measuring auxiliary qubits 3 and 4, which identifies the erroneous qubit among A,1,2 and corrects it.

The foundational scheme from Section 3 protects the encoded state against channel noise. We now introduce a third party Charlie, as a controller to enable supervised communication. The protocol starts by distributing a maximally entangled three-qubit GHZ state among the sender Alice, the receiver Bob, and the controller Charlie. The overall controlled dense coding protocol with integrated error correction is depicted in Figure 2.

**Step 1:** The GHZ state is a maximally entangled three-particle state, and can be expressed as follows

(5) $${|\Phi \rangle }_{ABC}=\frac{1}{\sqrt{2}}{\left(|000\rangle +|111\rangle \right)}_{ABC}$$

where qubit *A* belongs to Alice, qubit *B* to Bob, and qubit *C* to Charlie. This tripartite entanglement allows Charlie to influence the communication between Alice and Bob through measurements on his particle. After sharing the GHZ state particles, Charlie performs a projective measurement on his qubit *C* using the following basis

(6) $$|+\rangle =\frac{1}{\sqrt{2}}\left(|0\rangle +|1\rangle \right),\;|-\rangle =\frac{1}{\sqrt{2}}\left(|0\rangle -|1\rangle \right)$$

After that, Charlie transmits the measurement result to Bob. Depending on the result, the remaining two-particle state held by Alice and Bob collapses accordingly [39]. If the result is $|+\rangle$, shown as Equation (6), the state collapses to

(7) $${|\Phi^{+}\rangle }_{AB}=\frac{1}{\sqrt{2}}\left({|00\rangle }_{AB}+{|11\rangle }_{AB}\right)$$

If the result is $|-\rangle$, described by Equation (6), the state collapses to

(8) $${|\Phi^{-}\rangle }_{AB}=\frac{1}{\sqrt{2}}\left({|00\rangle }_{AB}-{|11\rangle }_{AB}\right)$$

**Step 2:** Alice then applies the corresponding Pauli operation, encodes the logical qubit into three physical qubits via the three-qubit repetition code, and sends them through a bit-flip channel where each qubit independently flips with probability 1−p.

**Step 3**: Upon receiving the three physical qubits, Bob introduces two auxiliary qubits (3,4) each initialized in the state $|0\rangle$. Then, he performs a nondestructive error diagnosis by indirectly measuring the stabilizers of the repetition code through controlled operations. Let φ denote the possibly erroneous state of the three received qubits (A,1,2). The state before diagnosis is then written as the product of this received state and the two auxiliary qubits

(9) $$|\Psi^{0}\rangle =|\varphi \rangle ⊗{|0\rangle }_{3}⊗{|0\rangle }_{4}$$

Bob applies two $C_{NOT}$ gates that both target the same auxiliary qubit 3, where the first gate is controlled by qubit *A* and the second by qubit 1.

(10) $$|\Psi^{1}\rangle =C_{NOT}\left(A,3\right)C_{NOT}\left(1,3\right)|\Psi^{0}\rangle$$

This operation records the parity of qubits *A* and 1 in auxiliary qubit 3. Thus, it measures the stabilizer $Z_{A}Z_{1}$ without disturbing the encoded quantum information. Similarly, Bob uses the second auxiliary qubit 4 to measure the parity of qubits *A* and 2

(11) $$|\Psi^{2}\rangle =C_{NOT}\left(A,4\right)C_{NOT}\left(2,4\right)|\Psi^{1}\rangle$$

After the above operations, the system reaches the final state $|\Psi^{2}\rangle$, in which auxiliary qubit 4 encodes the parity of qubits *A* and 2. This completes the parallel stabilizer measurement process. Bob then performs projective measurements on both auxiliary qubits in the basis of the states $|0\rangle$ and $|1\rangle$

(12) $$\left(s_{1},s_{2}\right)=\langle Z_{3}\rangle ⊗\langle Z_{4}\rangle$$

The outcome for two-bit measurement $\left(s_{1},s_{2}\right)$ constitutes the error syndrome, which provides crucial information about the location and potential bit-flip errors that may have occurred during transmission. For example, consider a bit-flip error on qubit *A*. This error is represented by the $\sigma_{x}$ operation acting on that qubit. The ideal logical state is

(13) $$|\psi_{L}\rangle =\frac{1}{\sqrt{2}}\left({|000\rangle }_{A12}+{|111\rangle }_{A12}\right)$$

After the error on qubit *A*, the state becomes

(14) $$X_{A}|\psi_{L}\rangle =\frac{1}{\sqrt{2}}\left({|100\rangle }_{A12}+{|011\rangle }_{A12}\right)$$

We now examine the effect of the error on the stabilizer measurements. Taking the first stabilizer $Z_{A}Z_{1}$, the expectation value for the state after an error is given by

(15) $$\langle Z_{A}Z_{1}\rangle =\left({\langle 100|}_{A12}+{\langle 011|}_{A12}\right)Z_{A}Z_{1}\left({|100\rangle }_{A12}+{|011\rangle }_{A12}\right)$$

Since $Z|0\rangle =+|0\rangle$ and $Z|1\rangle =-|1\rangle$, we find

(16) $$Z_{A}Z_{1}|100\rangle =\left(-1\right)\cdot \left(+1\right)|100\rangle =-|100\rangle$$

(17) $$Z_{A}Z_{1}|011\rangle =\left(+1\right)\cdot \left(-1\right)|011\rangle =-|011\rangle$$

From the calculation above, we obtain $\langle Z_{A}Z_{1}\rangle =-1$, which means that the eigenvalue is −1 and corresponds to the syndrome bit $s_{1}=1$ [40]. For the second stabilizer $Z_{A}Z_{2}$

(18) $$Z_{A}Z_{2}|100\rangle =\left(-1\right)\cdot \left(+1\right)|100\rangle =-|100\rangle$$

(19) $$Z_{A}Z_{2}|011\rangle =\left(+1\right)\cdot \left(-1\right)|011\rangle =-|011\rangle$$

Thus $\langle Z_{A}Z_{2}\rangle =-1$, which gives the syndrome bit $s_{2}=1$. In conclusion, a bit-flip error on qubit *A* yields the syndrome $\left(s_{1},s_{2}\right)=\left(1,1\right)$.

**Step 4**: Based on the measurement error syndrome $\left(s_{1},s_{2}\right)$, Bob applies the corresponding correction to restore the encoded state. The correction operation corresponding to each error syndrome is given in Table 1.

As shown in Table 1, the corresponding correction operator can be expressed as follows

(20) $$U_{c}\left(s_{1},s_{2}\right)=X_{A}^{s_{1}s_{2}}⊗X_{1}^{s_{1}\left(1-s_{2}\right)}⊗X_{2}^{\left(1-s_{1}\right)s_{2}}$$

This compact form applies a $\sigma_{x}$ operation only to the faulty qubit. For example, the syndrome (1,1) activates the term $X_{A}^{1}$, correcting an error on qubit *A*. Following error correction, Bob can recover the original logical qubit by applying the inverse of the encoding circuit. The encoding operation is unitary, and its inverse decodes the logical state. The $C_{NOT}$ gate satisfies $U_{CN}^{2}=I$, so applying it twice returns the qubits to their original state, and consequently the decoding circuit is identical to the encoding circuit

(21) $$|\psi_{l}\rangle =C_{NOT}\left(A,1\right)\cdot C_{NOT}\left(A,2\right)\cdot U_{c}|\varphi \rangle$$

**Step 5:** Bob now holds the recovered qubit *A* and his original qubit *B*. He performs a Bell-state measurement on these two qubits to decode the two classical bits. This protocol follows the bit-flip channel model from Section 3, and its error-correction success probability is the same as given in Equation (4). After successful error correction, Bob can perfectly decode the two classical bits, giving an average information $I_{total}=2P_{c}=2\left[p^{3}+3\left(1-p\right)p^{2}\right]$. This shows that although the controller enables supervised communication, the protocol’s resistance to channel noise is still determined by the error-correction performance of the three-qubit repetition code. In the maximally entangled limit, our protocol achieves the full dense coding capacity of two bits per transmitted qubit, which is the standard capacity for maximally entangled states [41].

Compared with the two-party error-correction scheme of Luo et al. using a maximally entangled Bell state [37], our protocol in this section introduces a three-qubit GHZ state and a third-party controller Charlie, thereby extending the original scheme to a controlled three-party framework. This enhancement improves system controllability, as Charlie’s measurement can authorize or deny the communication by projecting the shared GHZ state onto different Bell states between Alice and Bob [42]. The integration of error correction with supervisory control thus provides not only transmission reliability but also a governance mechanism for authentication and authorization in quantum networks [43,44].

## 5. Probabilistic Dense Coding with Partially Entangled States

**Figure 3:**
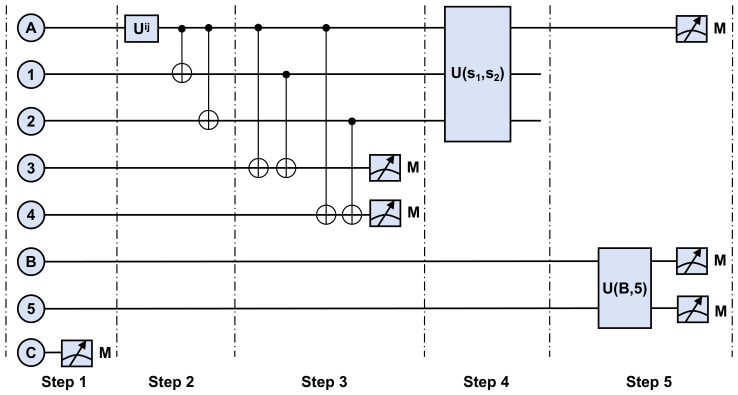
Quantum circuit for the error-corrected controlled dense coding protocol with partially entangled states and probabilistic decoding. Alice’s encoding and Bob’s error correction are the same as in Figure 2. The addition is the probabilistic decoding stage. After successful error correction, Bob introduces an auxiliary qubit 5 and applies a unitary U(B,5) to discriminate the four non-orthogonal states.

Some dense coding protocols are typically analyzed under the assumption of maximally entangled states. In practice, however, decoherence inevitably degrades these states into partially entangled ones [45]. To enhance the feasibility of the protocol, we extend it to partially entangled states, which are a more accessible resource for quantum communication [46]. This leads to a controlled probabilistic dense coding scheme for partially entangled states [47,48]. Based on the framework of Figure 2, the complete quantum circuit of the extended protocol is presented in Figure 3. The shared state is now partially entangled, and Bob’s decoding stage is supplemented by a unitary operation *U* on his qubit *B* and an auxiliary qubit 5. This operation is designed to probabilistically distinguish the four non-orthogonal encoded states. When the discrimination succeeds, the system collapses to a set of orthogonal states for a perfect Bell-state measurement.

**Step 1**: The protocol begins with a partially entangled three-qubit state

(22) $$|\Psi \rangle =a{|000\rangle }_{ABC}+b{|111\rangle }_{ABC}$$

where qubit *A* belongs to Alice, qubit *B* to Bob, and qubit *C* to Charlie. The coefficients *a* and *b* are real and satisfy ${\left|a\right|}^{2}+{\left|b\right|}^{2}=1$ with $\left|a\right|\geq \left|b\right|$. Charlie performs the same projective measurement as in Section 3 on his qubit *C* in the $|+\rangle ,|-\rangle$ basis defined in Equation (6) and communicates the result to Bob. This measurement collapses the two-particle state shared by Alice and Bob into one of the following partially entangled Bell states. If the result is $|+\rangle$, shown as Equation (6), the state collapses to:

(23) $${|\Phi^{+}\rangle }_{AB}=a{|00\rangle }_{AB}+b{|11\rangle }_{AB}$$

If the result is $|-\rangle$, described by Equation (6), the state collapses to:

(24) $${|\Phi^{-}\rangle }_{AB}=a{|00\rangle }_{AB}-b{|11\rangle }_{AB}$$

**Step 2**: In the following, we assume Charlie’s measurement outcome is $|+\rangle$. The case of $|-\rangle$ is analogous and leads to similar results. Alice’s encoding follows the same procedure as Step 2 in Section 4. She applies the corresponding Pauli operation to her qubit *A* based on her two-bit information, then encodes it into three physical qubits (A,1,2) using the three-qubit repetition code. The initial shared state is now partially entangled as given by Equation (23), whereas in Section 4 it was maximally entangled.

**Step 3**: Bob’s error diagnosis and correction follow Steps 3 and 4 in Section 4. Upon receiving the three encoded qubits, he introduces auxiliary qubits, performs stabilizer measurements to detect bit-flip errors, and applies the corresponding correction. After error correction, he recovers Alice’s original logical qubit *A*.

**Step 4**: Following the successful error correction and recovery of Alice’s original logical qubit *A*, Bob proceeds to the final decoding stage. He now possesses two qubits, the recovered qubit *A* and his own qubit *B*. Assuming Charlie’s measurement outcome was |+〉 and that Bob has been classically informed of this result, the entangled state shared between Alice and Bob before encoding is given by Equation (23). After Alice applies one of the four Pauli operations $\left\{I,\sigma_{x},\sigma_{y},\sigma_{z}\right\}$ to her qubit, the two-qubit state becomes one of the following four states:

(25) $${|\phi_{0}\rangle }_{AB}=a|00\rangle +b|11\rangle \to 00$$

(26) $${|\phi_{1}\rangle }_{AB}=a|00\rangle -b|11\rangle \to 01$$

(27) $${|\phi_{2}\rangle }_{AB}=a|10\rangle +b|01\rangle \to 10$$

(28) $${|\phi_{3}\rangle }_{AB}=a|10\rangle -b|01\rangle \to 11$$

Bob introduces an auxiliary qubit 5 initialized in ${|0\rangle }_{5}$ and applies a joint unitary operation *U* on the composite system of qubits *B* and 5 to probabilistically distinguish the four states [49,50,51]. Given the condition $\left|a\right|\geq \left|b\right|$, the unitary operation *U* is given by:

(29) $$U\left(B,5\right)=\left(\begin{array}{cccc} \frac{b}{a} & -\sqrt{1-{\left(\frac{b}{a}\right)}^{2}} & 0 & 0\\ \sqrt{1-{\left(\frac{b}{a}\right)}^{2}} & \frac{b}{a} & 0 & 0\\ 0 & 0 & 1 & 0\\ 0 & 0 & 0 & 1 \end{array}\right)$$

The operation *U* is designed so that

(30) $$U\left({|\phi_{j}\rangle }_{AB}⊗{|0\rangle }_{5}\right)=\sqrt{P_{s}}{|\psi_{j}\rangle }_{AB}{|0\rangle }_{5}+\sqrt{1-P_{s}}{|\chi_{j}\rangle }_{AB}{|1\rangle }_{5}$$

where ${|\psi_{j}\rangle }_{AB}$ is a set of mutually orthogonal states satisfying $\langle \psi_{j}|\psi_{k}\rangle =\delta_{jk}$ that can be perfectly distinguished by a subsequent projective measurement on qubits *A* and *B*, while ${|\chi_{j}\rangle }_{AB}$ are the residual states that arise when the discrimination fails and are generally non-orthogonal. The parameter $P_{s}$ is the success probability of the discrimination. For either of Charlie’s measurement outcomes, the success probability of unambiguous discrimination of the four states is [49,51]

(31) $$P_{s}=2{\left|b\right|}^{2},{\left|b\right|}^{2}<\frac{1}{2}$$

This expression for $P_{s}$ is consistent with the optimal success probabilities derived in probabilistic dense coding and quantum state discrimination [48]. Thus, the performance of the protocol depends on the initial entanglement. The weaker the entanglement, the lower the success probability, where the degree of entanglement is characterized by $\left|b\right|$. In the maximally entangled case where $\left|a\right|=\left|b\right|=1/\sqrt{2}$, we have $P_{s}=1$ and the scheme reduces to the deterministic protocol of Section 4. In the opposite limit where $\left|b\right|\to 0$, the state becomes a product state and $P_{s}\to 0$, making perfect decoding impossible.

After applying $U\left(B,5\right)$ shown as Equation (29), Bob measures the auxiliary qubit. The measurement outcome determines the subsequent decoding path. If the result is $|0\rangle$, which occurs with probability $P_{s}$, the discrimination succeeds. The system collapses into one of the orthogonal states ${|\psi_{j}\rangle }_{AB}$. Bob then performs a projective measurement on qubits *A* and *B* that distinguishes these orthogonal states perfectly, thereby recovering Alice’s two-bit classical information. If the result is $|1\rangle$, which occurs with probability $1-P_{s}$, the discrimination fails. The system collapses into one of the residual states ${|\chi_{j}\rangle }_{AB}$. Although Bob cannot perfectly distinguish all four $|\phi_{j}\rangle_{AB}$ in this branch, he can still extract partial information. As defined in Equations (25)–(28), the states $|\phi_{0}\rangle$, $|\phi_{1}\rangle$ correspond to messages with the first bit 0, while $|\phi_{2}\rangle$, $|\phi_{3}\rangle$ correspond to the first bit 1. By distinguishing whether the state belongs to the subset $\left\{\phi_{0},\phi_{1}\right\}$ or $\left\{\phi_{2},\phi_{3}\right\}$, Bob can recover the first bit of the message, which corresponds to one bit of information [52,53]. The average information obtained in the failure branch is therefore $I_{fail}=1$.

## 6. Performance Analysis

In Section 5, we obtained the success probability $P_{s}=2{\left|b\right|}^{2}$ for the probabilistic decoding stage, which depends on the initial entanglement. However, the overall protocol performance must also account for the bit-flip channel noise affecting the error-correction stage. Based on Equation (4), the probability of successful error correction is $P_{c}=p^{3}+3\left(1-p\right)p^{2}$. Thus, the total probability of perfectly decoding two classical bits becomes

(32) $$P_{total}=P_{c}\cdot P_{s}=\left[p^{3}+3\left(1-p\right)p^{2}\right]\cdot 2{\left|b\right|}^{2}$$

**Figure 4:**
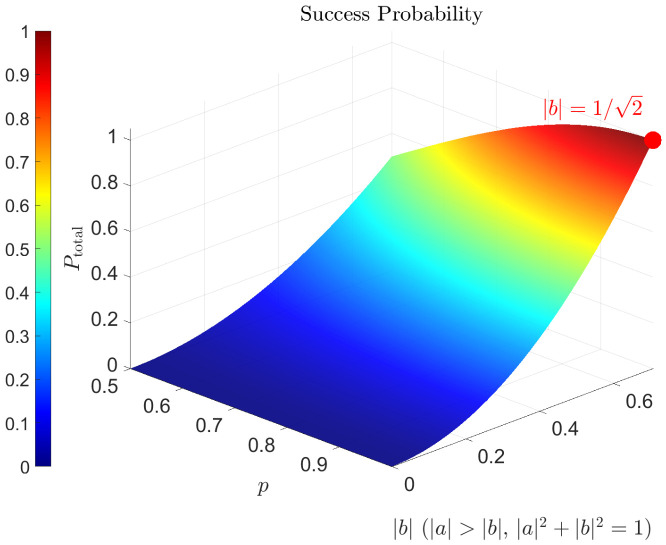
Success probability $P_{total}$ versus the channel quality *p* and the entanglement parameter *b*.

Figure 4 shows the dependence of $P_{total}$ on the channel quality *p* and the entanglement parameter *b*. $P_{total}$ increases monotonically with *p* and *b*, attaining its maximum value of 1 only at $\left|b\right|=\frac{1}{\sqrt{2}}$ and p=1.

In the probabilistic decoding protocol, the average information $I_{total}$ is defined as the weighted average of information obtained in both successful and failed scenarios:

(33) $$I_{total}=P_{c}\cdot \left[P_{s}\cdot I_{success}+\left(1-P_{s}\right)\cdot I_{fail}\right]$$

where $P_{s}=2{\left|b\right|}^{2}$ with ${\left|b\right|}^{2}\leq \frac{1}{2}$ is the success probability of the discrimination. Here $I_{success}=2$ bits and $I_{fail}=1$ bit as discussed in Section 5. Substituting these values yields

(34) $$I_{total}=P_{c}\cdot \left[P_{s}\cdot 2+\left(1-P_{s}\right)\cdot 1\right]=\left[p^{3}+3\left(1-p\right)p^{2}\right]\cdot \left(1+2{\left|b\right|}^{2}\right)$$

**Figure 5:**
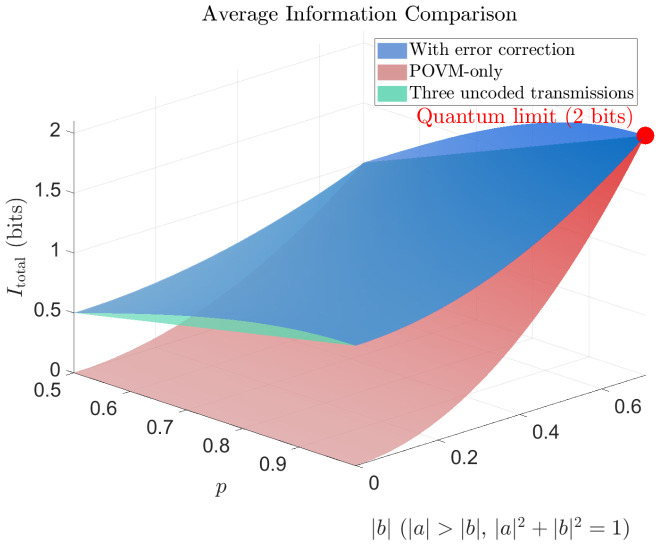
Comparison of the average information $I_{total}$ among three configurations as functions of the channel quality *p* and the entanglement parameter *b*: the proposed error-corrected protocol, the POVM-only scheme without error correction, and three uncoded transmissions.

To compare and highlight our proposed protocol performance under comparable resource constraints, we include two baselines in Figure 5. The first is three uncoded transmissions without error correction. For three independent transmissions, the average information can be evaluated by considering the cases of three, two, and one successful transmission

(35) $$I_{3\_successful}=C_{3}^{3}\cdot p^{3}\cdot 3\left(1+2{\left|b\right|}^{2}\right)$$

(36) $$I_{2\_successful}=C_{3}^{2}\cdot p^{2}\cdot \left(1-p\right)\cdot 2\left(1+2{\left|b\right|}^{2}\right)$$

(37) $$I_{1\_successful}=C_{3}^{1}\cdot p\cdot {\left(1-p\right)}^{2}\cdot \left(1+2{\left|b\right|}^{2}\right)$$

Averaging over the three transmissions yields

(38) $$I_{uncoded}=\frac{1}{3}\left(I_{3\_successful}+I_{2\_successful}+I_{1\_successful}\right)=p\left(1+2{\left|b\right|}^{2}\right)$$

The second is the optimal POVM decoding for partially entangled states without error correction. For the four non-orthogonal encoded states defined in Equations (25)–(28), the corresponding POVM operators are constructed as

(39) $$M_{0}=\frac{1}{\sqrt{2}a}\left(a|00\rangle +b|11\rangle \right)\left(b\langle 00|+a\langle 11|\right)$$

(40) $$M_{1}=\frac{1}{\sqrt{2}a}\left(a|00\rangle -b|11\rangle \right)\left(b\langle 00|-a\langle 11|\right)$$

(41) $$M_{2}=\frac{1}{\sqrt{2}a}\left(a|10\rangle +b|01\rangle \right)\left(b\langle 10|+a\langle 01|\right)$$

(42) $$M_{3}=\frac{1}{\sqrt{2}a}\left(a|10\rangle -b|01\rangle \right)\left(b\langle 10|-a\langle 01|\right)$$

(43) $$M_{4}=I-\sum_{i=0}^{3}M_{i}M_{i}^{†}$$

Taking $|\phi_{0}\rangle$ shown as Equation (25) as an example, the success probability is

(44) $$P_{0}=\langle \phi_{0}\left|M_{0}^{†}M_{0}\right|\phi_{0}\rangle =2{\left|b\right|}^{2}$$

The same result holds for the other three states shown as Equations (26)–(28). Thus, the success probability for each of the four states is $2{\left|b\right|}^{2}$, which is independent of the channel noise. Including the channel transmission probability *p*, the average information for the POVM-only baseline is

(45) $$I_{POVM}=p\cdot 2\cdot 2{\left|b\right|}^{2}=4p{\left|b\right|}^{2}$$

As shown in Figure 5, our protocol consistently outperforms both baselines across the entire parameter regime. The improvement over the three uncoded baseline confirms that the performance gain arises from the quantum error correction mechanism itself, rather than merely from the increased number of channel uses. The improvement over the POVM-only baseline confirms that error correction provides additional benefit beyond what optimized measurements alone can achieve. These results demonstrate the effectiveness of integrating the three-qubit repetition code with probabilistic controlled dense coding.

Beyond the two baselines shown in Figure 5, there are other strategies to improve dense coding performance. More powerful codes, such as the Steane code or the Shor code, can correct both bit-flip and phase-flip errors simultaneously. However, they require substantially more physical qubits and more complex syndrome measurement circuits [54,55]. Entanglement purification offers another alternative. This approach typically requires at least two shared partially entangled pairs, which are locally processed and measured to distill a smaller number of higher-fidelity entangled pairs before encoding [56,57]. It consumes multiple copies of the entangled resource to achieve deterministic decoding, whereas our protocol operates with a single copy using probabilistic decoding and active error correction. A direct numerical comparison between our protocol and these strategies would require different resource assumptions and noise models, and is therefore left for future investigation.

## 7. Discussion

In this section, we discuss the potential of computational optimization for quantum protocols and the experimental progress in dense coding systems. For complex quantum communication protocols, when the unitary matrix is known, decomposing it into elementary gates can be challenging and the resulting circuit is not necessarily optimal. Our current protocol uses the three-qubit repetition code, whose encoding and decoding circuits can be constructed directly. However, if it is replaced with a more complex error-correction code, the circuit design becomes not straightforward. In such cases, computational methods offer a promising way to design quantum circuits. End-to-end optimization methods that directly search for circuit structures, rather than first deriving a unitary and then decomposing it, are more likely to find hardware-adapted or noise-tailored implementations. Simulated annealing has been successfully applied to automatically discover quantum circuits for simultaneous dense coding [58]. Similarly, genetic algorithms and machine learning techniques have also been used to explore automated protocol design [59,60]. These successes suggest that similar optimization strategies could assist in designing encoding and decoding circuits for complex codes, or even refine the circuits of the present protocol under specific noise models.

Quantum dense coding is a typical protocol in quantum communication and has been extended to multiparty and partially entangled scenarios [61,62,63]. Our protocol, which uses a partially entangled state as the initial resource, also belongs to this type. This three-party supervisory architecture can be extended to other multiparty quantum communication primitives, such as quantum information splitting, where a sender distributes quantum information among multiple receivers and all must cooperate for recovery [64]. Unlike such deterministic schemes, our protocol falls into the category of probabilistic dense coding. Compared with previous probabilistic schemes, our protocol incorporates the three-qubit repetition code to combat bit-flip errors, thereby achieving higher average information. A limitation of the current design is that it only corrects single bit-flip errors and does not handle phase-flip errors or multiple errors. For other noise models, the situation is more complex. Phase-flip errors are not directly correctable by the repetition code, but they can in principle be converted to bit-flip errors via Hadamard gates before encoding and after decoding. Phase-damping and depolarizing noise would further reduce the fidelity of the shared entangled state, thereby lowering the success probability of the probabilistic decoding stage [65]. Addressing these noise models would require either concatenated code or more powerful error-correcting codes, which we leave for future investigation.

On the experimental side, various platforms for dense coding have been explored, each with its own advantages and disadvantages. Linear optical implementations offer scalability but suffer from low success probabilities and require post-selection [66]. Cavity-QED setups achieve higher fidelity, but are more demanding in terms of equipment and control [67]. More recently, dense coding has been integrated into long-distance quantum networks, where performance is still limited by entanglement quality and memory times [61]. Implementing our probabilistic controlled dense coding protocol requires high-fidelity $C_{NOT}$ gates, accurate estimation of entanglement parameters via quantum state tomography, and sufficiently long coherence times to complete encoding, transmission, and decoding within a single coherence window. Meeting these requirements simultaneously is currently challenging, but relevant progress has been made on superconducting and trapped-ion platforms [68,69,70].

In general, the combination of algorithmic optimization, theoretical protocol design, and experimental advances is gradually overcoming the obstacles of noise and entanglement degradation. With continued effort, these developments are pushing quantum communication toward practical applications in noisy and resource-limited environments. Our protocol represents a step in this direction by integrating error correction with probabilistic controlled dense coding.

## 8. Conclusions

In this work, we have proposed a protocol for probabilistic controlled dense coding by using quantum error correction. The protocol operates under realistic conditions, taking into account both channel noise and the use of partially entangled states. Compared with conventional dense coding schemes without error correction, the proposed protocol offers three distinct advantages. First, it incorporates a three-qubit repetition code to correct bit-flip errors, improving transmission reliability. Second, it works with partially entangled states, which are more feasible to prepare and maintain in practice. Third, it includes a control mechanism that enables a third party to supervise the communication without compromising robustness against noise. The protocol outperforms the POVM-only and three uncoded transmissions baselines, achieving higher average information across the entire parameter regime. This improvement is captured by the expression for $I_{total}$ given in Equation (34), which accounts for both successful and failed cases. These results show that the protocol is suitable for practical quantum communication scenarios where entanglement resources are limited and channel imperfections are unavoidable. Future work may extend this framework to more general noise models, potentially contributing to the development of scalable quantum communication systems.

## Figures and Tables

**Table 1:** Correspondence between error syndromes and correction operations.

Error Location	Qubit State	Syndrome ($s_{1},s_{2}$)	Action
No error	$\frac{1}{\sqrt{2}}(|000\rangle +\left|111\rangle \right)$	(0,0)	I⊗I⊗I
Qubit *A*	$\frac{1}{\sqrt{2}}(|100\rangle +\left|011\rangle \right)$	(1,1)	$X_{A}⊗I⊗I$
Qubit 1	$\frac{1}{\sqrt{2}}(|010\rangle +\left|101\rangle \right)$	(1,0)	$I⊗X_{1}⊗I$
Qubit 2	$\frac{1}{\sqrt{2}}(|001\rangle +\left|110\rangle \right)$	(0,1)	$I⊗I⊗X_{2}$

## Data Availability

No datasets were generated or analyzed during the current study.
